# The Advantages of Connectivity Map Applied in Traditional Chinese Medicine

**DOI:** 10.3389/fphar.2021.474267

**Published:** 2021-03-11

**Authors:** Huimin Jiang, Cheng Hu, Meijuan Chen

**Affiliations:** ^1^ School of Medicine and Holistic Integrative Medicine, Nanjing University of Chinese Medicine, Nanjing, China; ^2^ CAS Key Laboratory of Nutrition, Metabolism and Food Safety, Shanghai Institute of Nutrition and Health, University of Chinese Academy of Sciences, Chinese Academy of Sciences, Shanghai, China; ^3^ The First Clinical Medical College, Nanjing University of Chinese Medicine, Nanjing, China

**Keywords:** connectivity map, traditional Chinese medicine, Chinese herbal compound, synergetic mechanism, TCM repurposing

## Abstract

Amid the establishment and optimization of Connectivity Map (CMAP), the functional relationships among drugs, genes, and diseases are further explored. This biological database has been widely used to identify drugs with common mechanisms, repurpose existing drugs, discover the molecular mechanisms of unknown drugs, and find potential drugs for some diseases. Research on traditional Chinese medicine (TCM) has entered a new era in the wake of the development of bioinformatics and other subjects including network pharmacology, proteomics, metabolomics, herbgenomics, and so on. TCM gradually conforms to modern science, but there is still a torrent of limitations. In recent years, CMAP has shown its distinct advantages in the study of the components of TCM and the synergetic mechanism of TCM formulas; hence, the combination of them is inevitable.

## 1 Introduction

CMAP is a database in which mRNA expression levels of cells treated with different small molecule drugs are stored according to the degree of upregulation or downregulation compared with the control group. After the query signature (mRNA expression level was significantly changed) was found from the drug or disease, the connectivity scores of each drug molecule in the database ranging from −1 to 1 (from completely negative correlation to completely positive correlation) were available by querying the Connectivity Map. Finally, the known molecules were selected to do further research according to the scores (Gao et al., 2019). The five elements of the CMAP database are genomic signatures, cell lines, perturbagens, concentration and duration of treatment, and pattern-matching strategy. According to the principle that a few cells, low cost, high yield, and enough complexity to provide rich description, the first-generation Connectivity Map chose mRNA expression assayed on DNA microarrays as genomic signatures (Lamb et al., 2006). The perturbation in mammalian cell culture is more easy and economic to generate data. 164 disparate small molecules approved by FDA represent different bioactive tools to perturb cells. The dose sensitive to the result was explored through a subset of compounds across a range of concentrations. The duration of treatment is obliged to veritably reflect signals related to the direct mechanism. Instead of the traditional hierarchical clustering (Subramanian et al., 2005), the database employs new pattern-matching strategy based on the Gene Set Enrichment Analysis (GSEA) (Boratyn et al., 2006). In this article, we look back at the process of CMAP optimization in the last 20 years since the emergence of CMAP. In addition, we aim to summarize the articles published in the past ten years on the application of CMAP in traditional Chinese medicine (TCM) research and emphasize the advantages of CMAP in it.

## 2 The Optimizations of Connectivity Map

CMAP began with Daniel D. Shoemaker, who searched for the function of open reading frames (ORFs) in *yeast* by molecular bar-coding strategy using mutant *yeast* strains tagged with unique sequences and hybridizing with oligonucleotide array to amplify the signal in 1996 (Shoemaker et al., 1996). But few cellular functions possess the corresponding simple phenotypes to be applied. In 2000, Timothy R. Hughes established the genome database of 300 *S. cerevisiae* (with characteristic gene mutation, uncharacteristic ORF gene mutations, and treatment of known molecular target drugs) for gene function classification and discovery (Hughes et al., 2000). This idea was proposed as a database of reference expression profiles by a simple comparison of the most similar patterns of its gene expression profiles. In the same way, they applied it to discover the function of drugs and states of diseases. The pioneer work could be traced to Justin Lamb who first demonstrated that the Connectivity Map concept was indeed viable through majorization of the potential problem and recovery of known and new connections in 2006 (Lamb et al., 2006).

In recent decades, CMAP has become comprehensive with more drug molecules in the database and further optimized data processing methods. The publicly funded CMAP increased FDA-approved small molecules by 164–1,309. It also provides an interactive website and an online tool to conduct CMAP queries against the chemical reference catalogs (http://www.broadinstitute.org/cmap) (Qu and Rajpal, 2012). SSCMap (statistically significant connections' map) is a Java application designed to make statistically significant connections between a user-supplied gene signature and the 6,100 core reference profiles based on the Broad Institute expanded dataset (Zhang and Gant, 2009). Quite a few new types of CMAP have emerged, such as Functional Module Connectivity Map (FMCM), long noncoding RNA (lncRNA) Connectivity Map, and Drug directionality Map (DMAP). Functional Module Connectivity Map was devised for the discovery of repurposed drug compounds for systems treatment of complex diseases especially cancer (Xue et al., 2014). The core of FMCM is to construct condition-specific function–function networks (FFNs) to identify highly expressed hub genes before querying CMAP. In this way, higher hit rates around 65% on effective drugs against early tumorigenesis in colorectal cancer were presented. Shortly before, mRNA expression was transformed to lncRNAs as genomic signatures to connect small molecules, genes, and diseases, which is called lncRNA Connectivity Map (Yang et al., 2017). Different from traditional CMAP, lncRNAs as potential biomarkers play a key role in various biological processes and mechanisms. Drug directionality Map is an exhaustive drug-protein Connectivity Map constructed *in silico*, which makes up for the limitation of Connectivity Maps on data coverage. The database not only stores drugs for certain diseases, but contains directed drug-to-protein effects and effect scores to make drug repositioning efforts successful (Huang et al., 2015).

## 3 The Recent Achievements Utilizing Connectivity Map

To put that in perspective, CMAP has been widely used in pharmacogenomics, including identification of novel phenotypic relations for disease treatment, for drug repurposing and for studying drug combinations (Musa et al., 2018).

On the one hand, exploring the connections between drugs is facile. For example, the agonist and antagonist of same substance can be directly shown through the connectivity score, so that we can identify the drugs with common mechanisms of action, even though structurally diverse hERG (Human Ether-a-go-go-Related Gene) inhibitors are prone to be enriched through transcriptional response similarity (Brody et al., 2013). A class of drugs with common activity can be recovered by CMAP, which can predict the target path and even discover the unknown mechanism for small molecule drugs with unknown characteristics. Andrea M. Brum *et al.* analyzed gene expression profiles in hMSCs (human bone marrow mesenchymal stem cells) differentiated into osteoblasts and obtained query signature. They found parbendazole matched with the expression changes in osteogenic hMSCs as a candidate of bone stimulating compound. It further revealed the human osteogenic pathway by which parbendazole can induce osteogenic differentiation by combining cytoskeleton changes and the increase of bmp-2 activity (Brum et al., 2015). Withaferin A, calcium folinate, and amylocaine were underlying osteogenic drugs through stimulating both ALP activity and mineralization in hMSCs in light of positive correlation (Brum et al., 2018).

On the other hand, the link between drugs and diseases is equally important. For instance, the particular query signature directly from a disease or indirectly from a known drug efficacious to the disease can be used to filter other potential drugs for the disease through CMAP. Owing to multiple databases with massive gene expression profiles on CRC (colorectal cancer), CMAP analyzed a combined gene with a distinction of 148 genes to filtrate 10 candidate compounds, including irinotecan and etoposide (Wen et al., 2015). Similarly, it has been found that histone deacetylase inhibitors can inhibit the activity of hepatocytes and select the prognostic phenotype of HB cells (hepatoblastoma cells) by changing gene expression, which plays a synergistic role in combination with cisplatin (Beck et al., 2016). Drug candidates to restrain cigarette smoke-induced inflammation were kaempferol and bethanechol via CMAP (Vanderstocken et al., 2018). The difference is that the query signature here is from the contrast between the healthy and the smokers. Beyond that, the CMAP has shown promise in the treatment of cystic fibrosis (CF) (Malcomson et al., 2016), lymphoma (Luo et al., 2018), Huntington's disease (Smalley et al., 2016), breast cancer (Fang and Zhang, 2017), Hirschsprung disease (Xiao et al., 2018), and gastric cancer (Zhang et al., 2018) on account of identifying compounds and combination therapies.

Furthermore, CMAP serves to identify the novel therapies for existing drugs. Leptin has been dismissed as the main treatment for obesity with resistance. Amantadine, an FDA-approved antiviral and anti-Parkinson agent, is associated with a monocyte-macrophage-like differentiation of HL60, U937, and Kasumi-1 myeloid leukemia cell lines by inducing vitamin D receptor (VDR) expression (Manzotti et al., 2015). On account of Celastrol, a small molecule extracted from the roots of *Thunder of God Vine* plant and found to act as a sensitization of leptin through CMAP, leptin combined with it can be used to treat obesity with leptin deficiency (Liu et al., 2015). Nowadays there are many other staggering applications. It is critical to recover apoptosis subnetwork of the generalizable cell death pathways triggered by drug. CMAP can help restore the network (Yu et al., 2015). CMAP succeeds to carve out early prediction models of adverse drug reactions (ADRs) rendering the potential safety risks visible (Wang et al., 2015). What's more surprising is that RNAi and CRISPR technologies are evaluated by CMAP (Smith et al., 2017).

## 4 Application of Connectivity Map in Traditional Chinese Medicine

So far, the CMAP technology has been applied in a wide range of TCM studies. The application can be categorized in regard to their purpose of study. Specifically, CMAP in TCM has been used to discover the molecular mechanism of TCM, to interpret synergistic mechanism of TCM formula, to elucidate the veracity of some TCM for certain disease treatment, and even for TCM repurposing (Zhang et al., 2016).

### 4.1 The Principle of Connectivity Map of Traditional Chinese Medicine

**FIGURE 1:**
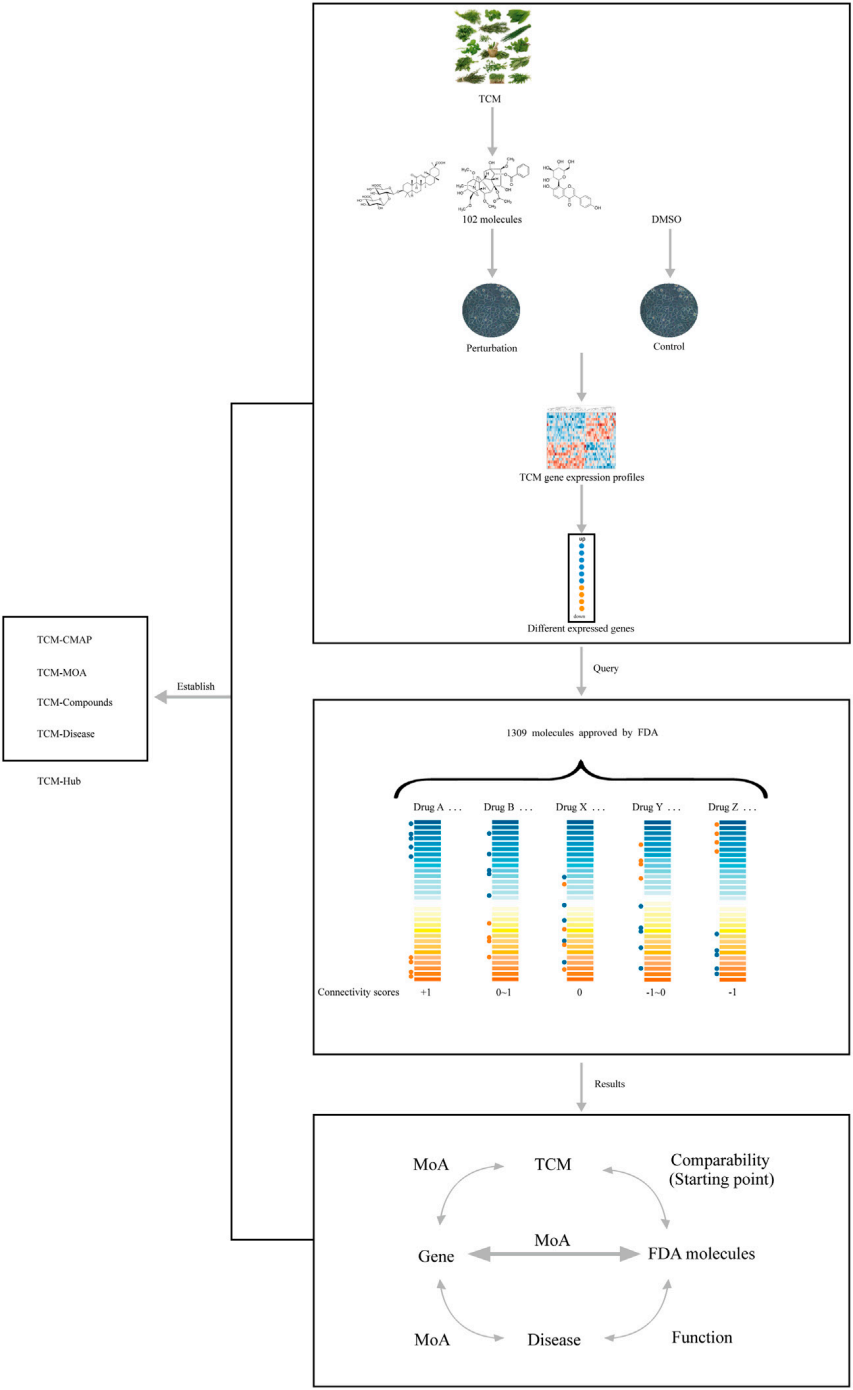
Simplified overview of the principle and route of Connectivity Map applied in TCM. In the first step, cells treated with 102 representative Chinese medicines molecularly were calibrated with the control group, and gene expression profiles were obtained by microarray technology. Then, different expressed genes were available to query the latest CMAP so connection scores were acquired with certain algorithm. Based on the results above, comparability between TCM and corresponding small molecules was the starting point. With that, the relationship between TCM, genes, and diseases has chance to be further analyzed. Finally, a TCM Hub database is established by integrating the results.

Although more and more meaningful results proved the necessity of CMAP for TCM research, only a few small molecule drugs approved by FDA in CMAP are components of TCM. There is a lack of systematic TCM-CMAP. Over the past few years, Weidong Zhang's laboratory has performed microarray analysis and fluorescence quantitative detection on MCF7 cells treated by 102 representative small molecules of TCM. In addition, each group was treated with DMSO as control, and the two groups were divided during the algorithm treatment to correct the results caused by the discrepancy in gene chip. Finally, the differential expression probe was selected as the query signature and input CMAP. The query result was obtained according to the same principle as the usual CMAP (Lv et al., 2017). On the basis of them, Minjae Yoo *et al.* query the 102 TCM components with gene expression signatures using the latest connectivity graph CLUE web application (https://clue.io/). The query results comprise 84 CMAP classes, and the MoAs (the mechanism of actions) for these TCM components are divided into seven categories based upon the classification of the inhibitors or activators as well as clustering analysis. TCM component clusters are four correspondingly. These results are stored to establish TCM Hub (Yoo et al., 2018). TCM Hub that is a connection diagram resource is freely available in http://tanlab.ucdenver.edu/TCMHub. It functions as a TCM-CMAP to query the MoAs of TCM, active ingredients, and Chinese medicine molecules associated with certain diseases (Figure 1).

### 4.2 Discovering Active Ingredients and MoAs of Traditional Chinese Medicine

Berberine (BBR) is an isoquinoline alkaloid known for its anti-inflammatory, lipid-regulating, anticancer, antidiabetic, antibacterial, antiparasitic, and fungicidal activities (Ayati et al., 2017). The multiple pharmacological effects of BBR are derived from its diverse molecular targets, so it takes a lot of work to study its MoAs (mechanism of action) by traditional methods, while CMAP can efficiently locate its various MoAs as a whole. Lee K. H *et al.* obtained molecules with high connectivity score by querying CMAP, top of which were protein synthesis inhibitors, mTOR, or HDAC inhibitor. With further querying the STTICH database and experimental verification, they found that potential MoAs of BBR were inhibition of global protein synthesis and the basic activity of AKT, induction of endoplasmic reticulum stress, and autophagy and acetylation of HDAC6 substrate a-tubulin (Kuhn et al., 2014; Lee et al., 2014). This provides a direction for further research on the specific pharmacological mechanism of BBR. Likewise, Yuan Quan *et al.* also predict the pharmacological mechanisms of curcumin (a typical isolated natural drug), including anti-inflammatory, anti-infective, and neuroregulation effect. Most of them were supported by experimental observations (Quan et al., 2014). Cinobufotalin is a compound extracted from the skin of dried *toads* and has a broad spectrum of antitumor activity, but the molecular mechanism is unknown. Jie Li *et al.* searched for matches in CMAP and found that cinobufotalin and miconazole had similar molecular mechanisms, with neuroactive ligand-receptor interactions and calcium signaling pathways being the key to their effects (Li et al., 2019).

The most comprehensive TCM researched through CMAP is probably *Salvia miltiorrhiza Bunge*. As a medicinal model plant, Danshen Transcriptional Resource Database (DsTRD) has been constructed for the reference of other researches (Shao et al., 2016). And compound Danshen dropping pill (DSP) is of great benefit in the treatment of atherosclerosis. Wei Zhou *et al.* first identified 10 candidates for active ingredients in it (Zhou et al., 2016a). And then Chao Lv found that tanshinone IIA (Tan IIA) (the main active ingredient derived from Danshen) and mTOR inhibitors, Hsp90 inhibitors, PKC inhibitors, and PI3K inhibitors have positive correlation in regard to gene expression characteristics, thus further demonstrating the antitumor mechanism of Tan IIA. Overall, Tan IIA may restrain activity of total protein kinase C (PKC) and Ras/MAPK pathway. In addition, it also significantly inhibited the PI3K/Akt/mTOR signaling pathway and induced cell cycle arrest and autophagy (Lv et al., 2018).

### 4.3 Exploring Synergistic Mechanism of Traditional Chinese Medicine Formula

The efficacy of TCM formula that consists of two or more herbs is the result of the synergistic effect of various natural compounds (Yuan et al., 2017). However, there is no strong evidence to fully prove the synergistic effect of Chinese herbal medicine. Some opponents assert that TCM formula is more likely to work as a placebo than to have more active ingredients. Inversely, most clinical cases show that TCM formula is more effective than single drug in the treatment of some chronic diseases or diseases with complicated causes (Zhou et al., 2016b). CMAP technology can appropriately reveal the synergistic effect of some TCM formula based on multiactive components and multitarget mechanism. At the same time, it can account for the magic that TCM formula suitable for a certain “ZHENG” is also efficacious for other different diseases in the perspective of western medicine (Zhang and Wang, 2014). Through the analysis of difference in the RNA expression of leukocyte in patients with qi-deficiency-blood-stasis syndrome before and after treatment with Fuzheng Huayu capsule, CMAP query found that Fuzheng Huayu capsule possesses antihyperglycemia, antihyperlipidemia, antihypotension, anti-inflammatory, and other effects. Its potential mechanism may involve Ca^2+^ related pathway (Yu et al., 2012). The four main active components of *Salvia miltiorrhiza Bunge* are tanshinone IIA, salvianic acid A sodium, protocatechuic aldehyde, and salvianolic acid B. Lv et al. applied the algorithm of random walk with restart (RWR) to compute the cardiovascular effect scores of the mixture of the four components and each single component separately. The mixture of the four components obtained a high effect score of 0.72 and the Z-score of 4.958, which was much higher than that of any single component. It suggested that the synergy among the four components is significantly associated with the effects of Danshen on cardiovascular disease (Lv et al., 2017). QiShenYiQi (QSYQ), a Chinese medicine, is composed of *Astragalus propinquus Schischkin* (Huangqi), *Salvia miltiorrhiza* (Danshen), *Panax notoginseng* (Sanqi), and *Dalbergia odorifera* (Jiangxiang). Query labels were identified from the rat model of myocardial infarction treated with QSYQ, and complex pathways of QSYQ's synergistic effect on myocardial infarction were delineated through compound-target-pathway network (Li et al., 2014).

### 4.4 Confirming or Predicting Traditional Chinese Medicine-Diseases

The ultimate goal of all research is to help the treatment of clinical diseases. The link between diseases and herbal medicines has been a focus of research. Recently, Xuetong Chen *et al.* took 189 disease gene expression profiles as pathological characteristics and 502 herbal medicine profiles as disturbance information and integrated the data to obtain the optimal combination of diseases based on this strategy (Chen et al., 2018). When CMAP was mentioned above to identify candidate drug for certain diseases, the obesity drug candidate Celastrol (the root extract of *Thunder of God Vine*) was introduced (Liu et al., 2015). Biochemical and pharmacological experiments indicated that the water-soluble polysaccharide MDG-1 extracted from *Radix Ophiopogonis* had strong effect of hypoglycemic and weight loss and was also a candidate drug for obesity-related metabolic diseases (Wang et al., 2014). In the same way, we could use CMAP to confirm the connection of TCM and corresponding diseases at the genetic level. For example, the gene expression profile of MCF7 cells treated with Si-Wu-Tang was put into CMAP, and the result disclosed that the score of estrogens was high, and the score of estrogen receptor antagonist was low. This falls into line with the fact that Si-Wu-Tang does well in gynecological diseases (Wen et al., 2011). In addition, CMAP and functional network approach using *in vitro* cell lines can be used to evaluate the pharmacological effects of drugs with *in vivo* systems. Kim *et al.* analyzed the microarray information of HepG2 cells treated with ESB (an extract of *Scutellaria baicalensis*) and input it into CMAP to obtain pharmaceutical profile, namely, the correlation set of small molecular drugs in CMAP. The pharmaceutical profiles of db/db mouse liver and human diabetic liver were obtained by the same method. Enrichment scores from human diabetic liver correlated positively with those of db/db mouse liver but inversely with those of ESB-treated HepG2 cells. Then common functional modules were established through functional network analysis to predict therapeutic effect of ESB on human diabetes finally (Kim et al., 2017). It is hoped that this method is of service to predict the pharmacological effects of herbs *in vivo* system by using cell lines *in vitro*.

### 4.5 Repurposing Traditional Chinese Medicine

Drug repurposing refers to seeking out undiscovered roles for existing drugs by dint of certain computational methods (De Bastiani et al., 2018). There are two mainstream methods: one is that structure determines properties paradigm; the other is based on omics data, especially the whole genome expression database (Sun et al., 2017). CMAP belongs to the latter, which makes it possible to reposition drugs by systematically comparing drug-related gene expression profiles. Dudly *et al.* compared the expression characteristics of IBD (inflammatory bowel disease) genes from public microarray data with those of 164 drug compounds to infer novel therapeutic relationships between drug–disease changes. Among the top-ranked drugs, in addition to the corticosteroid prednisone for traditional treatment of IBD, there was the antiepileptic drug topiramate, which was found to be more effective in subsequent IBD rat experiments (Dudley et al., 2011). In the same way, TCM has an array of functions to be excavated. For example, the most recent study of snake venom used functional genomics and concatenation mapping to recharacterize the biological activity of the *Bothrops jararaca* venom of the South American *pit viper*, suggesting that it may be useful in the treatment of neuropsychiatric and cardiovascular diseases (Nicolau et al., 2018). Drug developers can further research and develop drugs with potential clinical benefits. It takes billions of investment dollars and an average of about 9–12 years to bring a new drug to the market (Dickson and Gagnon, 2004). Investment in drug development has risen steadily, while the number of new drug approvals has stagnated. Compared to new drug discovery, CMAP as a computational drug repositioning may reduce the time and cost of drug discovery in preclinical and phase I trials, although repositioning of drugs may still present challenges after phase II trials (Li et al., 2016).

## 5 The Advantages of Connectivity Map Applied in Traditional Chinese Medicine

Zhou Zhongying, a master of Chinese medicine, once said that “the recession of traditional Chinese physician could be put down to traditional Chinese medicine.” It is foundational and crucial to pore through the cause of TCM. At present, the trend of TCM research is to extract single compound for further molecular biology study. The key to this approach is to find the effective single compound. As mentioned above, CMAP can be used to find the active ingredients of TCM, so it is of benefit to provide a direction for the extraction of single compound. However, traditional medical concept of TCM and modern pharmacology give us quite different feeling. Providing that we make use of the idea of single target and single component of western medicine, it is difficult to reflect the systematicness and synergism of TCM (Jiang, 2005). The synergistic therapeutic effect of TCM is derived from the complex interactions between a variety of bioactive ingredients in herbal medicines or herbal formulations. CMAP can reflect the synergistic effect of TCM through changes in gene expression. Moreover, compared with the low throughput of pharmacology and toxicology, CMAP has the advantages of being comprehensive, of high throughput, and accurate. The Broad Institute released the Library of Integrated Network-based Cellular Signatures (LINCS) which cover a vastly broader range of cell types and perturbagens (20,413 small molecules and 22,119 genetic perturbagens), along with the inferred expression of a further 21,305 probes (Keenan et al., 2018). Confronting with these challenges, new technologies stand out from the crowd, such as TCM proteomics, network pharmacology, and herbgenomics.

TCM proteomics seeks for potential targets through the differences in protein expression after the treatment of TCM or analyzes the differences in proteins from different parts of Chinese herbal medicine to find active components (Le et al., 2016). But most remain in the differentially expressed protein (DEP) levels, not clarifying internal mechanism (Suo et al., 2016). CMAP is committed to explore the MoAs of TCM. Wei Liu *et al.* used WGCNA to analyze the CMAP microarray dataset and isolated drugs into useful modules. They focused on the redundancy of gene function and gene-to-gene interactions that help predict drug behavior and identify MoAs (Liu et al., 2018).

After network pharmacology was firstly introduced into medical research by professor Andrew Hopkins L. (Hopkins, 2007), the researchers of Chinese medicine render it promising in collaboration with TCM, which broke the traditional model of “the single ingredient-the single target-the single disease” and made “disease-gene-targets-drug” integrate a network through the computer technology. Actually, it is quite a complex database with diverse genes, proteins, diseases, and drugs mutually crosslinked to form a whole (Li and Zhang, 2013). But network pharmacology of TCM lacks complex network pharmacology model to carry out plenty of work (Zhang et al., 2019). Beyond that, certain TCM with single active compound may well become complicated under guidance of network pharmacology theory. CMAP can simplify the study of TCM with complicated components and mechanisms and will not complicate the study of simple drugs.

The concept of herbgenomics was first proposed by Chen Shilin *et al.* (Hu et al., 2019). Herbgenomics in a narrow sense refers to beginning from the whole genome sequencing of TCM, gene mapping, and gene function analysis. It intends to establish systematic research of the original plant genome, with affirmative effect in distinguishing species of Chinese herbal medicine, looking for patterns, explaining biology essence of trueborn herb, and controlling quality of TCM. These are all basic studies of Chinese herbal medicine and do not involve disease or clinic. CMAP could confirm the connection of TCM and corresponding diseases at the genetic level from above. Cheng *et al.* compiled a set of 890 true drug-disease pairs from two different sources as a benchmarking standard. The disease signatures were generated using Gene Logic BioExpressTM system and the compound profiles were derived from the CMAP. The similarity scoring algorithm called eXtreme Sum (XSum) can achieve a fourfold enrichment at 0.01 false positive rate level, which confirms the high accuracy of CMAP in predicting association of drug and disease (Cheng et al., 2014).

## 6 Discussion

In the wake of the emergence of microarray technology and the wide application of gene expression profile in pharmaceutical research, CMAP, as a database connecting genes, drugs, and diseases, has been extensively used to elucidate the mechanism of action, discover potential drugs for diseases, and relocate new drugs (Musa et al., 2018). TCM as the gem of China is an indispensable part of people's healthy life. Compared with traditional pharmacology, toxicology, Chinese medicine chemistry, and other research methods, CMAP has the characteristics of high throughput and being automatic, rapid, and simple. Besides modern methods such as herbgenomics and network pharmacology, it is also outstanding relying on the advantage of being more comprehensive and efficient (Liu et al., 2018). For instance, network pharmacology is only appropriate for the analysis of complicated TCM, whereas some simple ones tend to be complicated and not effectively distinguished (Liang et al., 2014). CMAP can identify multicomponent and multitarget drugs from the gene level and can easily research simple drugs as well. In addition, gene expression profiles of 102 TCM ingredients were recently studied, and a small CMAP database of TCM was established for the first time as a general template for TCM research (Lv et al., 2017). Minjae Yoo *et al.* further classified the molecular mechanisms of these TCMs (Yoo et al., 2018). CMAP has succeeded in analyzing the molecular functions of TCM, the synergistic mechanism of TCM components, the multiple functions of some compounds, and the detoxification theory of TCM (Zhang et al., 2016). Nevertheless, CMAP still demonstrates the issues. One is the limitations of the database itself. For example, we need to distinguish between widely activated conserved genes and widely expressed genes from those that are authentically regulated by drugs. In particular, some low-expressed genes are susceptible to detection noise (Zou et al., 2017). Otherwise, many unrelated genes involved in the construction of “connectivity” may be ambiguous, so OneComp was applied in advance to screening for differentially expressed genes (DEGs) related to treatment (He et al., 2017). Furthermore, combining the data of different cell lines through the probabilistic model can basically ignore the influence of different cell line gene data (Parkkinen and Kaski, 2014). The other is the limitation of the drugs in question. Certain drugs are not enough to be studied through CMAP, such as ingredients that work only after long-term exposure *in vivo* and components whose target genes are not expressed in all types of cells, etc. In particular, established gene expression profiles for the study of TCM components are only 102 molecules (Lv et al., 2017). This method can only pore through the connections from the known corresponding relationship between TCM molecules and genes in the database, but it fails to find new targets or pathways. In future studies, more investigators will further expand the number of small molecules. These data will provide more possibilities for the study of the molecular mechanism of TCM and eventually establish its own database. A previous study has illustrated that about 5% of the tested hypothesis of drug–gene interaction is verified in Connectivity Map (Mears et al., 2017). In a word, this way of using CMAP to study TCM is promising and tortuous and arduous, which entails people to keep on studying with the help of bioinformatics and other modern scientific technologies. Connectivity Map is doomed to be a sword to promote the development of TCM.
